# Host-Parasite interaction between brown algae and eukaryote biotrophic pathogens

**DOI:** 10.1016/j.crmicr.2024.100306

**Published:** 2024-10-24

**Authors:** Anagha Santhosh, Sigrid Neuhauser

**Affiliations:** Institute of Microbiology, Universität Innsbruck, Innsbruck, Austria

**Keywords:** Brown algae, Pathogen interaction, Defence, Stress response, Pathosystem, Parasite

## Abstract

Brown algae belong to the class Phaeophyceae which are mainly multicellular, photosynthetic organisms, however they evolved independently from terrestrial plants, green and red algae. In the past years marine aquaculture involving brown algae has gained enormous momentum. In both natural environments and aquaculture, brown algae are susceptible to infection by various prokaryotic and eukaryotic parasites. While our understanding of host-parasite interactions in brown algae is gaining recognition, our understanding of how brown algae react to biotic stress remains incomplete. The objective of this review is to address research gaps in the field by providing a summary of what is already known about the response of brown algae to abiotic and biotic stress. The biology of eukaryotic zoosporic pathogens *Maullinia ectocarpii, Eurychasma dicksonii, Anisolpidium ectocarpii* is also discussed, as those parasites have been used in laboratory experiments to study diseases of brown algae. These studies often relied on parasites-infecting *Ectocarpus siliculosus* which has become a brown algal model organism to study host-pathogen interactions. Stress response in brown algae involves processes similar to hypersensitivity response, oxidative stress response, and activation of peroxidases, but also the production of blue fluorescent metabolites and deposition of β-1,3-glucan in the cell wall. Cell wall modification, expression of several defence related proteins, and secondary metabolite production also hold a crucial role in brown algal defence mechanism. Understanding host-pathogen interactions and the associated mechanisms is vital to discover strategies to control pathogens in the growing aquaculture sector.

## Highlights

• The defence strategies against pathogens, specifically the ones mediated by chloroplast proteins, have resemblances between plants and algae.
• Processes similar to hypersensitive response and oxidative burst are ubiquitous and seem to be a highly generalized stress response.
• Haloperoxidases are important stress response of brown algae against pathogens.
• Brown algal defence strategies include changes in gene expression, secondary metabolite production and activation of signalling cascades.

## Graphical abstract

**Figure:**
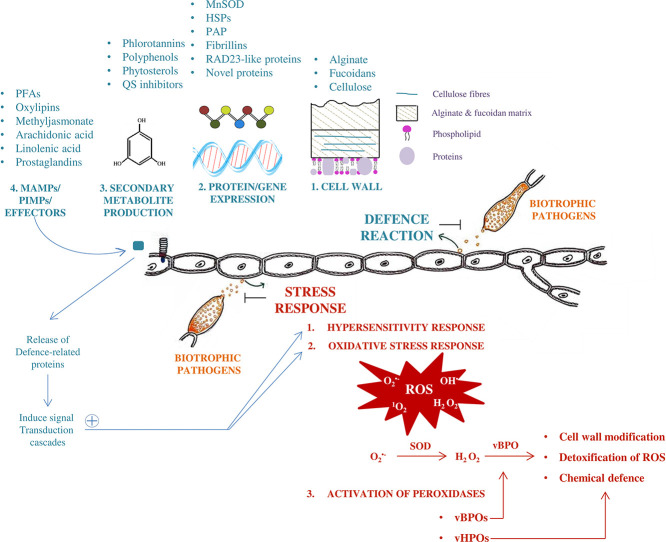

## Introduction

Algae are important ecosystem builders that drive key nutrient cycle processes and contribute to primary production in cold and temperate coastal seas (Dayton, 1985). Brown algae are photosynthetic multicellular organisms belonging to the Stramenopila which are part of the TSAR Supergroup (Charrier et al., 2008; Dittami et al., 2009, Burki et al., 2019). They are not only distant from plants and green algae but also from red algae and the Opisthokonta, which includes both fungi and animals (Dittami et al., 2009, Burki et al. 2019). Kelps are large sessile organisms and are of high ecological significance in the coastal ecosystem. (Ritter et al., 2014, Teagle et al., 2017) However, they are under threat from ocean warming and acidification (Smale, 2020) as well as from parasites and pathogens (Murúa et al., 2023). Many biological characteristics differentiate brown algae from most of the terrestrial plants, including their cell wall composition that comprises cellulose, alginic acid, and several polysaccharides, the main storage polysaccharide laminarin, the presence of phycodes (vesicular intracellular inclusions), chloroplasts with thylakoids arranged in three stacks and surrounded by a girdle lamella, the ability to accumulate iodine and to synthesise both C_18_ and C_20_ oxylipins (Dittami et al., 2009; Wehr, 2015; Beuder and Braybrook, 2023). Brown algae evolved differently from other photosynthesisers. Therefore, they possess several novel metabolic, cellular, physiological, and ecological traits (Charrier et al., 2008, Bringloe et al., 2020). All these traits influence how brown algae respond to biotic and abiotic stressors.

The main threats to global biomass production of brown algae are currently abiotic stressors and the resulting changes in community structures due to this changing environment (Teagle et al., 2017, Filbee-Dexter and Wernberg, 2018, Bringloe et al., 2020). Seaweeds are especially vulnerable to global environmental shifts due to their sessile nature and restricted dispersal of propagules (Wahl et al., 2015). Most brown algae inhabit the intertidal zones that are constantly exposed to natural environmental fluctuations in temperature, exposure to environmental pollutants, hypo and hyper-saline conditions, and oxidative stress (Dittami et al., 2009; Ritter et al., 2014). All these changes lead to physiological changes in brown algae (Dittami et al., 2009). An example for an abiotic stressor is copper stress that disrupts photosynthesis by competing for magnesium binding sites of chlorophyll molecules (Küpper et al., 2002b), and by triggering ROS accumulation (Zou et al.; 2015;Pinto et al., 2003; Yruela et al., 2000). Increased ROS level alters the cell structure, signalling cascades including the activation of oxylipin production (Ritter et al., 2008). Another frequent environmental stressor is changing salinity levels. Brown algae tolerate a wide range of salinity levels, with fluctuating saline conditions reducing their tolerance and affecting photosynthetic efficiency (Karsten, 2007).

The brown algae *E. siliculosus* was used for experimental, physiological, and -omics approaches in brown algae because of its filamentous, rapid growth, and its relatively small genome. It was also used for identifying and studying brown algal response to abiotic and biotic stress (Tonon et al., 2011). Findings obtained through research with *E. siliculosus* helped our understanding of brown algae stress response. It is also used to study pathogen interactions, and was used as host to generate transcriptomes of an increasing number of biotic parasites. Biotic stressors are a topic crucial for the future brown algal research because pathogens are expected to increase with the intensification of aquaculture. Therefore, having access to a brown algal model which allows studying biotic interactions will result in a better, more detailed understanding of host-parasite interactions (Cock et al., 2012, Murúa et al., 2023) but also more general biotic interactions between brown algae and microorganisms in controlled laboratory experiments. While abiotic stressors currently account for the majority of losses in marine aquaculture, there is an increasing recognition of the negative impact of biotic stressors on production and yield (Murúa et al., 2023).

Biotic stressors can be parasites, but there is a constant interaction between the brown algae and diverse microorganisms that are endo- and epiphytes of algae. Those interact as mutualists, commensals or parasites (Singh and Reddy, 2016, Markussen Bojorbaekmo et al., 2023). Among the primary biotic stressors, pathogens play a crucial role in affecting brown algae. Various eukaryotic parasites infect these algae, causing harm in their natural habitats and in aquaculture facilities (Gachon et al., 2017b, Murúa et al., 2023). The biology and diversity of the eukaryotic parasites of brown algae remain understudied, especially as they belong to taxonomically very different groups (Gachon et al., 2010; Neuhauser et al., 2011, Markussen Bojorbaekmo et al., 2023). The oomycete *Eurychasma dicksonii* was described as the most frequent eukaryotic pathogen of brown algae (Schwelm et al., 2018) and at least 45 taxa of brown algae are infected by this endoparasite (Müller et al., 1999; Sekimoto et al., 2008). Other parasites of brown algae include the oomycete *Anisolpidium ectocarpii,* the fungus *Chytridium polysiphoniae,* or the obligate biotrophic phytomyxid *Maullinia ectocarpii* (Charrier et al., 2008). *M. ectocarpii, A. ectocarpii* and *Eu. dicksonii* can be maintained in *E. siliculousus* in the lab, which allows to study these pathosystems in functional bioassays (Vallet et al., 2018) or for genomic studies.

By focusing on the pathogenicity of selected eukaryotic pathogens and the host response of infected brown algae, aim of this study is to improve our understanding of diseases of algae in both natural environments and commercial production systems. Understanding diseases of algae is crucial to optimize algal cultivation practices and to enhance resilience. This review is particularly timely given that these data are limited while there is a steady growth of the aquaculture sector. The changing environment will likely worsen the challenges posed by these pathogens, impacting ecological stability. This review provides a detailed overview of the infection strategies employed by several biotrophic pathogens that target brown algae. This is complemented by a summary of the stress reactions observed in brown algae, including hypersensitive response (HR), oxidative stress response, and the activation of peroxidases. Additionally, this review explores the specific pathogen defence mechanisms employed by the algae, such as restructuring their cell walls, regulating protein and gene expression, and synthesizing secondary metabolites. These data will provide crucial information to bridge the current research gap and lay the groundwork for future investigations.

## Biotrophic parasites of brown algae

Pathogenic bacteria, viruses, and eukaryotes constantly challenge macroalgae (Potin et al., 2002, Badis et al., 2019, Capistrant-Fossa et al., 2021, Egan et al. 2013, Lachnit et al., 2016), yet these organisms coexist with brown algae as part of a complex holobiont (Egan et al., 2013; del Campo et al. 2020, Bass et al., 2019). Besides pathogenic microorganisms, also parasitic brown algal endophytes such as *Laminariocolax* spp*.* and *Laminarionema* spp. can parasitise other brown algae (Bernard et al., 2019) These parasitic brown algae use an enzymatic penetration mechanism to enter their host (Heesch and Peters, 1999; Bernard et al., 2019). However, the molecular basis of the infection and kelp response to an infection with *Laminariocolax* spp. and *Laminarionema* spp. remain uncertain (Bernard et al., 2019). Many species of chytrid fungi have been described on brown algae in the monography “The aquatic phycomycetes” by Sparrow (1960), which is still the most extensive account on such species to date. One example is the chytrid fungus *Chytridium polysiphoniae,* which has a broad range of hosts including species like *E. siliculosus* and *E. fasciculatus* (Müller et al., 1999). However many of the species described by Sparrow in his book have no linked molecular and physical record, making it difficult to estimate the diversity of these parasites in modern taxonomic concepts but indicating a high diversity of such parasites.

**Fig. 1:**
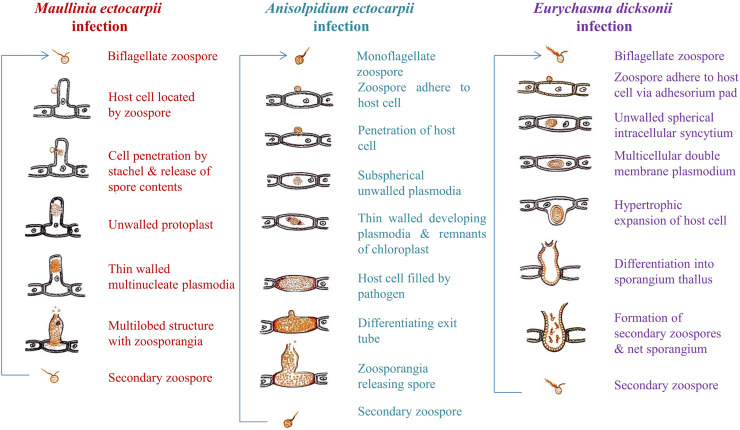
Infection mechanism of three biotrophic pathogens such as *Maullinia ectocarpii, Anisolpidium ectocarpii* and *Eurychasma dicksonii* in brown algae.

Microbial eukaryotic pathogens can have disastrous effects on aquatic plants, but we still poorly understand the factors that influence the spread of infection (Blake et al., 2017, Murúa et al., 2023). The study of spread, infection, and development under natural conditions would be beneficial, but it is incredibly challenging to achieve. Therefore, having access to laboratory cultures of eukaryotic parasites will help to increase our understanding of this field. Model pathosystems can be used to study interactions between biotrophic pathogens and their hosts through targeted and controlled laboratory experiments (Garvetto et al., 2023, Hittorf et al., 2023, Badstöber et al., 2020). This has already improved our understanding of those interactions, allowed to build parallels’ to related plant pathogens for which more data are available. In the following sections we will describe three zoosporic parasites of brown algae such as *M. ectocarpii, A. ectocarpii,* and *Eu. dicksonii,* because these three parasites have been used in controlled laboratory studies and it is important to understand their similarities and differences (Fig. 1).

***Maullinia ectocarpii*** belongs to the Phytomyxea (Rhizaria, TSAR group) which is an exclusively parasitic group of protist parasites of flowering plants and stramenopiles (Maier et al., 2000; Murúa et al., 2017; Neuhauser et al., 2011, 2014; Schwelm et al., 2018). Phytomyxea are obligate biotrophs, requiring living hosts to complete their life cycle (Bulman and Neuhauser, 2017). The active and growing phytomyxid inside of the host cells is a multinucleate plasmodium, zoospores are the propagation stage, and resting stages surrounded by a thick cell wall ensure survival under adverse conditions or during the absence of a host (Bulman and Neuhauser, 2017). Parasites of brown algae belong only to the order Phagomyxida, with currently two described species in the genus Maullinia. *M. ectocarpii* infects various brown algal genera such as *Ectocarpus* spp*., Macrocystis* spp*., Acinetospora* spp., *Durvillea* spp. while *M. braseltonii* infects giant kelps of the genus *Durvillea* spp. (Maier et al., 2000; Goecke et al., 2012; Blake et al., 2017). *Maullinia ectocarpii* was established in stable co-cultures with *E. siliculosus*, which allows for this species being used for functional bioassays (Vallet et al., 2018, Theodorou and Charrier, 2021).

Researchers have not yet described the full life cycle for either of the two species, but they have described the sporangial part (or primary infection) for *M. ectocarpii* while the sporogenic (or secondary infection) was only observed in *M. braseltonii*. This understanding of the life cycle is important, as the restriction to the sporangial part defines the limitations of microcosm experiments involving *M. ectocarpii* and suitable algal hosts. Although there is still a lack of microscopic evidence, we can assume that *M. ectocarpii* will form resting spores and induce galling in suitable hosts, because sequences of *M. ectocarpii* were generated from galls on the fronds of *Durvillea* spp. (Blake et al. 2017). After releasing zoospores into the environment, phytomyxids locate a suitable host to start the sporangial part of their life cycle. They retract their flagella when they encounter the host and encyst. A tubular cavity (Rohr) holding a bullet-like structure (satchel) which allows to penetrate the host cell wall is formed (Keskin and Fuchs, 1969; Aist and Williams, 1971). The unwalled uninucleate protoplast of the pathogen is “injected” into the host cell by the increased turgor pressure in the encysted zoospore (Bulman and Neuhauser, 2017). This protoplast then develops into a zoosporangial plasmodium (Aist and Williams, 1971; Braselton and Miller, 1975; Miller and Dylewski, 1983), where synchronous mitotic divisions result in a multinucleate plasmodium, which later cleaves into a thin-walled multicelled structure ultimately differentiating into zoospores (Dylewski and Miller, 1983). In cultures of *M. ectocarpii* and suitable hosts only this part of the life cycle can be observed to date.

The mechanisms that are mediating the switch to the sporogenic phase are not yet understood, but they start when secondary zoospores infect a compatible host, which in *Maullinia* spp. include the fronds of *Durvillea* spp. In those gigant kelps the spores invade the medulla tissue, where multinucleate plasmodia develop into resting spores (Goecke et al. 2012). The fronds of *Durvillea* suffer from hypertrophied host cells and galling because of this process. The multinucleate plasmodia exhibit a size increase compared to the sporogenic phase, and develop into sturdy resting stages that facilitate prolonged survival in the environment. The production of these thick-walled resting spores marks the end of the sporogenic phase, along with the onset of meiosis and appearance of cleavage furrows (Dylewski and Miller, 1984).

***Anisolpidium ectocarpii* (Oomycota, Stramenopiles)** belongs to the pathogenic oomycetes. Oomycetes affect most organisms as pathogens including algae, molluscs, crustaceans, plants, nematodes, fungi, fishes, and mammals (Murúa et al., 2020). The cosmopolitan genus *Anisolpidium* spp. contains the first identified example of anteriorly uniciliate oomycete (Gachon et al., 2017b), which is notable as stramenopiles usually possess two heterokont flagella. *A. ectocarpii* is an obligate biotrophic parasite of brown algae and can complete its infection cycle in a wide range of algal hosts belonging to more than twenty species from nine orders of brown algae. Suitable hosts include *Ectocarpus* spp., *Hincksia* spp., and *Macrocystis pyrifera* (Gachon et al., 2017b). Researchers have reported most members of this genus from field material and direct observation rather than based on cultures with all members sharing a similar infection cycle (Gachon et al. 2017b). The zoospores of *A. ectocarpii* are intramatrical-globular, later pyriform, and slightly clavate. The zoospores are hyaline with coarsely granular protoplasm. These spores remain highly motile for about 15 to 45 minutes, after which they get progressively sluggish and finally stop moving altogether (Karling, 1943). They penetrate the host cell and emerge as a subspherical unwalled plasmodia inside the host cell (Gachon et al., 2017b).

While the pathogen grows, the nucleus, cytoplasm, plastids, and other organelles of the host cell degrade rapidly and chestnut brown remnants of the chloroplasts can be observed at the end of the life cycle (Karling, 1943; Gachon et al., 2017b). Kashiyama et al (2019) reported the presence of reddish brown to dark-brown globules in algal cultures and was found to have formed during the dismantling of chloroplasts which is associated with chlorophyll catabolic cascade (CACC). The cells displayed thus chloroplast dismantling starting with the disappearance of the chlorophyll autofluorescence, followed by shrinking and darkening of chloroplasts (Kashiyama et al., 2019). The developing plasmodia form a thin cell wall and the host cell is successively filled by the pathogen (Gachon et al., 2017b). Studies conducted by Karling (1943) reported that this pathogen does not cause hypertrophy or distortion of the host cell. Instead, a reduction of cell diameter by half was observed when compared to the normal cell.

***Eurychasma dicksonii* (Oomycota, Stramenopiles)** is another example of parasitic oomycetes. The geographically widespread oomycete, *Eu. dicksonii* is an intracellular pathogen with a wide host range of over 45 algal species and can also tolerate a temperature range of 4 to 23 ° Celsius (Tsirigoti et al., 2014). *Eu. dicksonii* has mainly been recorded in wild populations of *Pylaiella littoralis*, although it has a wide host range which allows it to infect a wide variety of brown algae (Müller et al., 1999; Küpper and Müller, 1999). The estimated time for the complete developmental cycle of this pathogen is between 13 to 16 days. The small unwalled thalli appear approx. 8 to 10 dai occupying almost half the host cell volume and gradually expanding, resulting in the so-called ‘foamy stage’ where the parasite cytoplasm is abundant with vacuoles (Petersen, 1905; Sekimoto et al., 2008). The thallus continues to expand laterally as it turns into a sporangium (Sekimoto et al., 2008). Zoospores released from the mature thallus move towards and encyst the host filaments at their surface (Tsirigoti et al., 2014). Then, the pathogen's cytoplasm enters the algal cell by penetrating its wall, resulting in a multinucleate double membrane plasmodial thallus (Tsirigoti et al., 2014). The host cell wall at the infection sites thickens with an accumulation of β-1,3- glucans (Luna et al., 2011), and the mature thallus later develops a cell wall, causing a hypertrophic expansion of the algal host and then emerges as a sporangium (Tsirigoti et al., 2014; Strittmatter et al., 2016). The sporangial wall comprises three layers: a thin outer dense granular coat, an intermediate fibrillar layer, and a thicker electron lucent inner zone. This inner layer seems to be absent in the secondary cyst. The cell wall thickens slowly over stages of encystment (Tsirigoti et al., 2014).

Primary cysts are differentiated in a layer that is tightly linked to the sporangial wall. They ultimately rupture, releasing the ovoid-bean shaped biflagellate zoospores via an open apical exit tube. The presence of both vacant and filled cysts results in the formation of a residual net structure called net sporangium which is a differentiating feature of *Eu. dicksonii* infection compared to other zoosporic parasites (Sekimoto et al., 2008). After finding host filaments, zoospores encyst and securely adhere to the cell wall by developing an adhesorium pad (Sekimoto et al., 2008). After being released from the host, the inner core of the rod is imposed to rupture the cell wall (Sekimoto et al., 2008; Tsirigoti et al., 2014) causing the formation of cup structure that holds the parasitic thallus encircled by the cytoplasmic debris of the disintegrating host, whereas the cell wall looks stretched and tapered (Sekimoto et al., 2008). The growth of vegetative thallus of the oomycete is characterized by synchronous nuclear divisions that occur repeatedly (Heath, 1980). Whereas the vegetative state is concluded as the nuclear divisions stop and a multinucleate syncytium transforms into a zoosporangium through cellularization (Brown et al., 1994).

## Stress response in brown algae

Knowledge on pathogen-induced stress response in macroalgae is scarce, but hypersensitive response, ROS generation, production of microbe-associated or pathogen-induced molecular patterns, and peroxidases, as known from plant pathogens seems to play a role (Cosse et al., 2009; Balint-Kurti, 2019; Mansoor et al., 2022, Fig. 2). Hypersensitivity response (HR) describes the rapid and localized death of cells challenged by different stressors including parasites (Heath, 2000; Weinberger and Friedlander, 2000; Tang et al., 2002). In Kelps, alginate-degrading bacteria and rot disease could induce HR related mechanisms (Wang et al. 2004, Meili, 1992). It appears that exposure to bacterial flagellin-based elicitors leads to the induction of HR and reactive oxygen species (ROS) (Wang et al., 2013). HR has been described as part of the response of *E. siliculosus* to *Eu. dicksonii.* HR is accompanied by the production of blue fluorescent metabolites and deposit β -1,3-glucan at the cell wall, activating programmed cell death (PCD) associated markers (Gachon et al., 2017a). A second mechanism of general defence of the host against eukaryote pathogens was the activation of autophagy of the host cells in the *E. siliculousus* - *A. ectocarpii* pathosystem (Gachon et al., 2017a; Murúa et al., 2020).

**Fig. 2:**
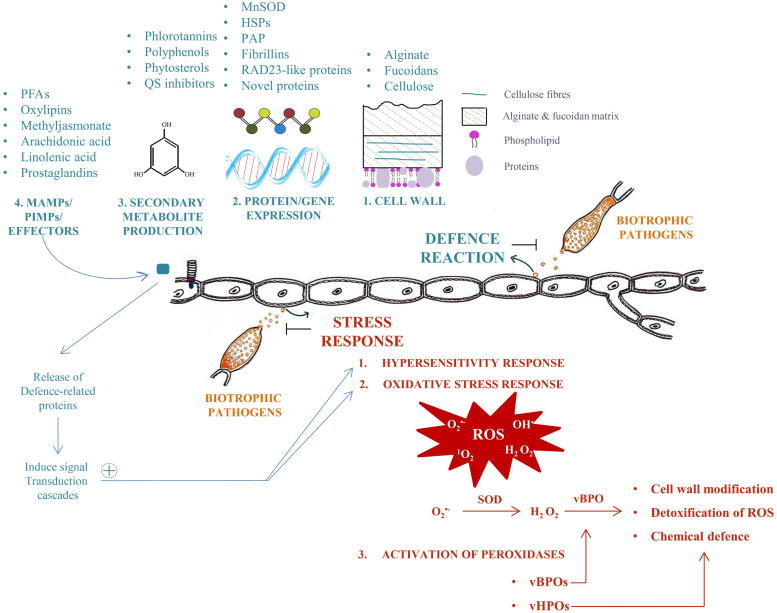
An overview of various defence mechanisms and stress response in brown algae when exposed to biotrophic pathogens. In blue are different processes related to active pathogen defence while in red more general stress response processes which are a part of the pathogen defence are listed.

Another ubiquitous response to biotic stress are microbe-associated molecular patterns (MAMPs; e.g. bacterial lipopolysaccharides) or pathogen-induced molecular patterns (PIMPs; e.g. oligoguluronates) which were shown to induce a response in macroalgae (Küpper et al., 2006; Dring, 2005; Weinberger, 2007). In *Laminaria digitata,* the ROS production was high enough to considerably slow down microbial growth (Weinberger and Friedlander, 2000). Therefore, ROS accumulation is a direct antimicrobial defence response of macroalgae, and ROS serves as promoter of more targeted downstream defence mechanisms like oxylipin accumulation and generation of halogenated secondary metabolites (Weinberger, 2007). Signal transduction between cells was also reported to be part of the algal defence against biotic stress (Cosse et al., 2009). Cells that are being in contact with pathogens are sending out waterborne signals that prime nearby individuals for rapid defence gene activation (Strittmatter et al., 2016), so an elicitor-induced defence mechanism generates a priming effect (Thomas et al., 2011).

As a common response to biotic stressors, macroalgae exhibit an increased rate of internally produced ROS (Mansoor et al., 2022). These oxygen radicals can be produced in chloroplasts, mitochondria, peroxisomes, plasma membranes, the endoplasmic reticulum, and the cell wall (Bischof and Rautenberger, 2012; Das and Roychodhury, 2014). Superoxide dismutase (SOD) is one of the primary defence of the cell against ROS (Bischof and Rautenberger, 2012), so it is not surprising that different SODs are involved in the stress response of diverse algae (Collén and Davison, 2001, Sáez et al., 2015, Liu and Wang, 2020). Two proteins, manganese superoxide dismutase (MnSOD) and vanadium bromoperoxidase (vBPO), are involved in ROS detoxification based on proteomic studies of *Eu. dicksonii* infected *E. siliculosus* (Strittmatter et al. 2016).

To detoxify ROS, peroxidases play a significant role in oxidative stress management (Bartsch et al., 2008; Bischof and Rautenberger, 2012). Haloperoxidases catalyse the halide ions by oxidation to hypohalous acid in the presence of H_2_O_2_. In *Laminaria,* these enzymes are located in the external cortical region of the thallus and particularly around the mucilaginous channels (Almeida et al., 2001). In *Saccharina latissima* and *L. digitata,* the areas where bromoperoxidase was active were only the blades (Jordan et al., 1991; Mehrtens and Laturnus, 1997). A rise in halomethane generation followed in response to oxidative stress, and the photosynthetic transport of electrons was inhibited (Palmer et al., 2005). In consequence, the increased H_2_O_2_ generation by the Melher reaction decreased the halogenation (Goodwin et al., 1997). Volatile halogenated organic compounds play critical roles in brown kelp effluxes, ensuring a rapid disposal of the ROS detoxification products (La Barre et al., 2010). These defence processes can have direct impact on the success of the infection of the host by the pathogens.

The best studied peroxidases are Vanadium-dependent haloperoxidases, which are the most prevalent stress markers in *Laminaria* spp. and *Ectocarpus* spp. (Crépineau *et al*. 2000; Roeder *et al*. 2005; Cosse *et al*. 2009). In brown algae, vanadium haloperoxidases and halides are crucial in oxidative detoxification, cell wall strengthening, and chemical defence. They play a role in the attachment of young sporophytes to their substrate but also are needed for the defence against epiphytic organisms and predation (La Barre et al., 2010). For the zoosporic pathogens discussed here especially the function in algal cell wall modification of vBPO is relevant (Cock et al., 2010; Strittmatter et al., 2016), because these pathogens enter their host through the cell wall. The mediated cross-linking of phenolic compounds and alginate in the presence of H_2_O_2_ (Salgado et al. 2009) can reinforce the host cell wall making it more difficult for the pathogens to enter the host cell. An involvement of vBPO during the infection process has been demonstrated with *M. ectocarpii* infections in *E. siliculosus* and *M. pyrifera* (Badstöber et al., 2020). Similar cell wall modifications have been triggered as a response to *Eu. dicksonii* infection of brown algae (Tsirigoti et al., 2013, 2014). However, different stressors induce a variable response of different haloperoxidase families (e.g. iodo- and bromoperoxidase) in *L. digitata* (Crépineau *et al*. 2000; Roeder *et al*. 2005; Cosse *et al*. 2009), so more targeted research on these proteins is needed to identify peroxidases crucial during infection.

## Defence mechanisms of brown algae against eukaryotic biotrophs

While stress response and pathogen defence share similarities, the latter are usually more targeted and specialised. The primary layer of defence of the host against pathogens is the cell wall, as this barrier has to be crossed by any pathogen prior to a successful infection event. Brown algal cell wall possesses various distinct characteristics (Beuder and Braybrook, 2023). Similar to terrestrial plants, cellulose is an integral part of the cell wall, but then alginates and fucoidans are the main building bricks of the brown algal cell wall (Cock et al., 2012; Deniaud-Bouët et al., 2017). Other compounds which occur in smaller quantities in the brown algal cell wall include proteins, halide compounds, and mixed linked glucans like β-3-linked glucans (Deniaud-Bouët et al., 2017; Raimundo et al., 2017; Salmeán et al., 2017). *Eu. dicksonii* invasion caused reinforcement of brown algal cell walls via the deposition of β -1,3-glucan, a analogous to the formation of callose (a β-1,3-glucan)-containing papillae in terrestrial plants (Underwood, 2012). This structural reinforcement at the site where the parasites try to enter is a rapid, yet critical defence strategy that putatively slows down pathogen penetration, while inducing more targeted defence mechanisms, such as gene expression changes and biochemical responses (Voigt, 2014; Murúa et al., 2020).

Another strategy to combat pathogens is the expression of defence-related proteins. Infection with *Eu. dicksonii* in *E. siliculosus* leads to an upregulation of 21 algal proteins, involving classical stress response proteins like MnSOD, heat shock protein 70 (HSP70), but also some yet uncharacterised proteins (Strittmatter et al., 2016; Mayer and Bukau, 2005). These uncharacterised proteins include chloroplast localized proteins, such as PAP/ fibrillins that make up major protein constituent of plastoglobules (lipoproteins structures) in plants. Plastoglobules consist of lipids and enzymes, and are linked to the chloroplast thylakoid membrane through a half-lipid bilayer where they serve as a central hub for stress-related signal transduction (Austin *et al*. 2006; Ytterberg *et al*. 2006; Bréhélin *et al*. 2007; Singh et al., 2010). The role of these plastoglobules in the specific defence against eukaryotic parasites is therefore likely. Another protein overexpressed in *E. siliculousus* infected with *Eu. dicksonii* was the RAD23-like protein which is a signalling molecule mediating enhanced protein degradation by the ubiquitin-26 proteasome mechanism. This is again linked to HSP70 conformation changes (Strittmatter et al., 2016) again hinting at a specific and targeted response against *Eu. dicksonii.* Overall, this study highlights that there are specific transcriptional changes happening upon infection which involve general host defence mechanisms but also pathogen specific changes.

A third layer of pathogen defence is the formation of bioactive secondary metabolites that serve as chemical defences against pathogens (Engel et al., 2006). In brown algae these metabolites include phlorotannins, polyphenol, and phytosterol (Hakim and Patel, 2020). Phlorotannins are unique to brown algae and comprise polyphenolic polar metabolites and a cell wall constituent (Amsler and Fairhead, 2005; Hakim and Patel, 2020) and play a major role in cytokinesis, for adhesion to substrate, and in wound sealing and healing (Schoenwaelder, 2002; Amsler and Fairhead, 2005; Schoenwalder and Clayton, 1998). However they are also reported to possess anti-herbivory and antibacterial properties (Wijesekara et al., 2010). Phlorotannins like phloroglucinol triacetate, diphlorethol pentacetate, and triphlorethol-A-heptacetate were observed in the brown algae *Cystophora congesa*, the extracts of which exhibited better antioxidant and cytotoxic properties (Yoon et al., 2008; Young e al., 2007), therefore these compounds are well characterised. Brown algae have been reported to possess immense antiparasitic activity against *Leishmania* spp., *Plasmodium falciparum* and *Tripanosoma brucei* (Fouladvant et al., 2011; Gallé et al., 2013; Perumal et al., 2018; Remya et al., 2022), much of it based on such secondary metabolites. Targeted studies following the production, type, and fate of such secondary metabolites as response to the infection of brown algae by parasites are lacking and will provide a promising field for study in the future.

Interactions within the holobiont of brown algae based on quorum sensing (QS) and similar processes, and eukaryotic pathogens have not been studied to date (Atkinson and Williams, 2009; Burgunter-Delamare et al., 2020). However it is known that brown algae can disrupt bacterial communication networks by generating QS inhibitors, impacting on biofilm formation and inducing changes in the gene expression of bacteria (González and Keshavan, 2006). As an example, *L. digitata* has been reported to produce hypobromous acid, which interferes with QS by disabling AHL (acyl-homoserine lactones) signals, a major component of QS in various bacteria (Borchardt et al., 2001). Similar external signals such as vBPO were shown to be produced as a response to infecting eukaryotic parasites (Badstöber et al., 2020). However, here we find another massive gap in our understanding of the interaction between brown algae, eukaryotic parasites, and the algal holobiont. It is possible that processes changing the nature or species composition of bacterial biofilms associated with brown algae also impact the success and severity of an infection with eukaryotic parasites.

The interaction between brown algae and pathogens often triggers complex signalling cascades in the host involving so-called microbe-associated molecular patterns (MAMPs) and pathogen-induced molecular patterns (PIMPs). MAMPs include parts of (harmful) microbes or compounds that are the result of microbial activity such as microbial cell wall compounds or lipopolysaccharides. These microbe-associated signals are recognised by the host and induce signalling cascades and often defence-related processes (Küpper et al., 2002a; 2009; Weinberger, 2007). PIMPs are defined as endogenously released products that are formed in response to the host defence against harmful microbes and reinforce these defences (Egan et al., 2014). These processes have been well established for brown algae like alginate-oligosaccharides induced oxidative burst in *L. digitata* that resulted in the accumulation of UV-fluorescent material, which is assumed to be defence associated aromatic products (Küpper et al., 2002a). Also signalling compounds like oxidized polyunsaturated fatty acids or oxylipins are thought to have a vital role in regulating macroalgae defence along with stress-related substances like hormones, arachidonic acid, linolenic acid, and prostaglandins (Potin et al., 2002; Bouarab et al., 2004; Küpper et al., 2009). These processes appear to be host specific, as so far proof of a universal chemical defence across different brown algae is lacking (Wiesemeier et al., 2008). Oxylipins for example did induce pathogen resistance in *L. digitata* but not in *E. siliculosus* (Zambounis et al., 2013). In general, brown algae have complex defence mechanisms against eukaryotic biotrophs. These mechanisms include strengthening cell wall structures, producing stress-response proteins, employing chemical defences, and utilizing advanced signaling pathways. The sophisticated defence mechanisms of algae serve as evidence of their remarkable ability to adapt and survive in the dynamic and harsh marine environment.

## Conclusion

The growing importance of marine aquaculture has led to an increased research focus on host-parasite interactions as intensification of farming procedures will likely result in increased disruptions through pathogens and parasites. Defence mechanisms such as HR, ROS, chloroplast-mediated defence strategies, and oxidative burst which are common response of angiosperm plants towards pathogens are present in a similar manner in brown algae. Therefore, building on our knowledge of plant pathology, expanding and characterising the function and regulation of these mechanisms in algae will offer interesting starting points for pathogen control and stress reduction of the algae. Breeding or engineering algae with enhanced production of example β-1,3-glucans, improved cell wall rigidity, or by selecting strains overexpressing haloperoxidases would be promising strategies to find strains with improved resilience against infections. Pathogen control and specific enhancements of resistance against pathogens will require more in-depth knowledge about specialised responses which include for example gene expression changes as a result of infection, the production of bioactive compounds, and understanding signalling cascades which protect the algae. Likewise, our understanding of elicitors or QS signals that induce a systemic priming effect is very limited, so clearly more targeted studies are needed to understand and address these factors in a systematic way in marine aquaculture. However, the research done to date could be used to identify early biomarkers of stress response and monitor their progression to be able to counteract pathogen infections at an early stage where the harvest can still be saved. Examples for such putative biomarkers are enhanced ROS production, monitoring the expression of defence-related genes, or monitoring the presence of specific metabolites like phlorotannins. An uptick in those markers could then serve as an early warning signal that a pathogen is present. Some basic concepts of phycopathology have currently been established, but targeted and focussed research on the parasite's molecular approach to interacting with their host is needed to provide a deep understanding of such pathogen systems and to ultimately develop efficient control strategies for pathogens in aquaculture and natural habitats.

## CRediT authorship contribution statement

**Anagha Santhosh:** Conceptualization, Writing – review & editing, Supervision, Project administration, Funding acquisition. **Sigrid Neuhauser:** Validation, Investigation, Data curation, Writing – original draft, Visualization.

## Declaration of competing interest

The authors declare that they have no known competing financial interests or personal relationships that could have appeared to influence the work reported in this paper.

## Data Availability

No data was used for the research described in the article.
